# Genome-wide identification, expression pattern and genetic variation analysis of *SWEET* gene family in barley reveal the artificial selection of *HvSWEET1a* during domestication and improvement

**DOI:** 10.3389/fpls.2023.1137434

**Published:** 2023-02-13

**Authors:** Wenhao Yue, Kangfeng Cai, Xue Xia, Lei Liu, Junmei Wang

**Affiliations:** ^1^ Key Laboratory of Digital Dry Land Crops of Zhejiang Province, Zhejiang Academy of Agricultural Sciences, Hangzhou, China; ^2^ National Barley Improvement Center, Hangzhou, China; ^3^ College of Advanced Agricultural Sciences, Zhejiang Agricultural and Forestry University, Hangzhou, China

**Keywords:** barley, SWEET, expression profile, haplotype, domestication

## Abstract

SWEET (Sugars Will Eventually be Exported Transporter) proteins, an essential class of sugar transporters, are involved in vital biological processes of plant growth and development. To date, systematical analysis of *SWEET* family in barley (*Hordeum vulgare*) has not been reported. In this study, we genome-wide identified 23 *HvSWEET* genes in barley, which were further clustered into four clades by phylogenetic tree. The members belonging to the same clade showed relatively similar gene structures and conserved protein motifs. Synteny analysis confirmed the tandem and segmental duplications among *HvSWEET* genes during evolution. Expression profile analysis demonstrated that the patterns of *HvSWEET* genes varied and the gene neofunctionalization occurred after duplications. Yeast complementary assay and subcellular localization in tobacco leaves suggested that *HvSWEET1a* and *HvSWEET4*, highly expressed in seed aleurone and scutellum during germination, respectively, functioned as plasma membrane hexose sugar transporters. Furthermore, genetic variation detection indicated that *HvSWEET1a* was under artificial selection pressure during barley domestication and improvement. The obtained results facilitate our comprehensive understanding and further functional investigations of barley *HvSWEET* gene family, and also provide a potential candidate gene for *de novo* domestication breeding of barley.

## Introduction

All terrestrial life depends on the photosynthesis, that plants produce carbohydrates (e.g. sugars, starch) in source tissues (mesophyll cells) and transport the carbon assimilates long-distance *via* the phloem sieve element-companion cell complex to sustain the growth and development of sink tissues, such as roots, flowers, fruits and seeds (Ruan, 2014). In most plants, sucrose is the principal carbohydrates transported *via* either symplastic or apoplastic pathway, in which sugar transporters play important roles (Chen et al., 2015a). SWEET transporters were firstly characterized in last decades (Chen et al., 2010), mostly mediating sugar efflux following the concentration gradient (Baker et al., 2012) and involving in essential biological processes, for example sucrose export from mesophyll cells to apoplast for phloem loading (Chen et al., 2012; Bezrutczyk et al., 2018), sugars transfer from seed coat and endosperm to embryo during the seed filling stage (Chen et al., 2015b; Wang et al., 2019), sucrose export from nectary parenchyma to extracellular space to recruit pollinating insects (Lin et al., 2014), etc. However, pathovar-specific effectors can hijack the sugar efflux system and target the promoters of specific *SWEET* genes to induce gene expressions, increasing sugar content in the invasion sites to fuel their growth (Antony et al., 2010; Chen et al., 2010). The conserved domain in SWEET is MtN3/Saliva, which consists of two units of three transmembrane helices (TMHs) separated by a less conserved TMH in plants (Han et al., 2017). The Arabidopsis *SWEET* genes are mainly clustered into four clades, with clade I, II and IV preferentially transporting monosaccharides and clade III disaccharides, respectively (Eom et al., 2015). The SWEET proteins exhibit substrate recognition and selectivity based on the size of the substrate-binding pocket and function in oligomerization manner (Han et al., 2017). Given the importance of *SWEET* genes in sugar allocations, many *SWEET* genes underwent artificial selection during crop domestication. Maize and rice *SWEET4*, which mediate the hexose transport across the basal endosperm transfer layer, the entry point of nutrients into the seed, were strongly selected during domestication to sustain the development of the large starch-storing endosperm of cereal grains (Sosso et al., 2015). The artificial selection of soybean *GmSWEET10a* drove the initial domestication of multiple seed traits, such as seed size, oil content and protein content (Wang et al., 2020).

With the rapid development of sequencing technology, more and more reference genomes were available, leading to genome-wide identification of *SWEET* genes in various species, such as Arabidopsis (*Arabidopsis thaliana*) (Chen et al., 2010), rice (*Oryza sativa*) (Yuan and Wang, 2013), soybean (*Glycine max*) (Patil et al., 2015), sorghum (*Sorghum bicolor*) (Mizuno et al., 2016), wheat (*Triticum aestivum*) (Qin et al., 2020). The barley genome with size around 5G is characterized by high content of repetitive elements and large pericentromeric regions that are virtually devoid of meiotic recombination, and the first chromosome level reference genome publication (Mascher et al., 2017) makes it possible to genome-wide analyze the *HvSWEET* gene family. Although barley *HvSWEET* family members were identified in previously study (Mascher et al., 2017; Qin et al., 2020), they were not systematically investigated. In this study, we comprehensively analyzed phylogenetic relationships, gene structures and conserved protein motifs, syntenic relationships, expression patterns of *HvSWEET* family genes, and found that the plasma membrane (PM) localized hexose transporter HvSWEET1a, highly expressed in aleurone tissue during seed germination, underwent artificial selection during barley domestication and improvement.

## Materials and methods

### Identification, characterization, and phylogenetic analysis of *HvSWEET* genes in barley

Barley *SWEET* candidate genes were identified by combinations of conserved protein domain and BLAST searching methods. The barley reference genome and protein sequences (Mascher et al., 2017) were downloaded from Phytozome (https://phytozome-next.jgi.doe.gov/). A total of 17 AtSWEET proteins and 21 OsSWEET proteins identified previously (Chen et al., 2010) were obtained from corresponding Arabidopsis (Cheng et al., 2017) and rice (Ouyang et al., 2007) files in Phytozome. The Hidden Markov Model (HMM) of the MtN3_slv domain (PF03083), downloaded from Pfam 35.0 (http://pfam-legacy.xfam.org/), was used to identify putative barley SWEET proteins using HMMER (http://hmmer.org/) with the “trusted cutoff and E-value < 0.01” as the threshold. The 17 Arabidopsis SWEET proteins were used as queries to carry out a BLASTP search in the barley protein sequences with the E-value < 1e^-5^ as the threshold using DIAMOND (Buchfink et al., 2021) (Version 2.0.11). The barley SWEET proteins, identified by both HMM and BLASTP methods, were manual checked and named in accordance with previously study (Mascher et al., 2017). The R package Peptides (Osório et al., 2015) (Version 2.4.4) was used to calculate the physical and chemical parameters of HvSWEET proteins, including protein length, molecular weight and theoretical pI. The Multiple sequence alignment of HvSWEET proteins was performed using MUSCLE program implemented in MEGA7 (Kumar et al., 2016) with the default parameters, and the phylogenetic tree was constructed by MEGA7 with the bootstrap of 1000 replications using neighbor-joining (NJ) method. The phylogenetic tree of SWEET family proteins of Arabidopsis, rice and barley was constructed using the same method without OsSWEET7d/LOC_Os09g08490, for short protein length (63 aa). The MEME software (Bailey et al., 2009) (Version 5.0.5) was used to investigate the conserved motifs of HvSWEET proteins with the parameters ‘-mod anr –nmotifs 10 –minw 6 –maxw 200’. The amino acid sequences of 10 conserved protein motifs were plotted using R package ggseqlogo (Wagih, 2017). The phylogenetic tree, conserved protein motifs and gene structures of *HvSWEET* genes were shown with iTOL (https://itol.embl.de/).

### Synteny analysis of SWEET family genes and construction of species tree

The reference primary protein files of Arabidopsis (Cheng et al., 2017), rice (Ouyang et al., 2007), maize (Jiao et al., 2017), sorghum (McCormick et al., 2018), barley (Mascher et al., 2017), and wheat (Zhu et al., 2021) were downloaded from Phytozome, and used to perform genome-wide syntenic analysis within barley or between barley and other plant species. Proteins of plant species were subject to homologous searching by DIAMOND BLASTP with the paramenters ‘–evalue 1e^-10^ –max-target-seqs 5’. The MCScanX (Wang et al., 2012) was used to deal with the BLASTP results to identify the gene duplication events and the synteny results were visualized by Circos (Krzywinski et al., 2009) (Version 0.69-8) or python version JCVI (https://github.com/tanghaibao/jcvi) of MCScan. The OrthoFinder (Emms and Kelly, 2019) was used to make gene family clustering. The orthogroups, containing three genes in wheat and only one gene in other plant species, were identified and the genes, with two wheat genes randomly discarded, were derived to assemble super genes to construct maximum-likelihood (ML) species phylogenetic tree using IQ-Tree (Nguyen et al., 2015) (Version 1.6.12) with the parameters ‘-m MFP -bb 1000’.

### Expression analysis of *HvSWEET* genes

To investigate the *HvSWEET* gene expression patterns, the RNA-seq raw sequencing data of 16 different barley developmental tissues (leaf, root, inflorescence, etc) (Mascher et al., 2017) and seed tissues during germination (embryo, aleurone, scutellum and grain) (Betts et al., 2017) were downloaded from NCBI, and filtered using fastp (Chen et al., 2018) (Version 0.12.4) with the default parameters. The high-quality cleaned reads were aligned to the barley reference genome (Mascher et al., 2017) with HISAT2 (Kim et al., 2019). Following alignments, raw counts for each gene were derived using featureCounts implemented in R package Rsubread (Liao et al., 2019), and normalized into the number of transcripts per kilobase of exon sequence in a gene per million mapped reads (TPM) with TMM method (Robinson and Oshlack, 2010). Heatmaps of *HvSWEET* gene expression profiles were generated using the R package ComplexHeatmap (Gu et al., 2016) (Version 2.10.0) based on the log_2_ (TPM +1) transformation or expression proportions of *HvSWEET* genes in that of the whole gene family calculated by TPM values.

### Haplotype and median-joining network analysis

The exome SNP data of 360 barley accessions (20 wild accessions, 166 landraces and 174 cultivars) with clearly known breeding history from previously published study (Bustos-Korts et al., 2019) were derived and used to analyze the genetic diversity of *HvSWEET* genes. The SNPs with missing data >10% or minor allele frequency (MAF) < 5% were filtered using VCFtools (Danecek et al., 2011) (Version 0.1.17), and then the missing data were imputated using the package beagle (Browning et al., 2018) (Version 5.4) with the default parameters. The SNPs in *HvSWEET* genes were extracted to perform haplotype analysis and annotated according to the barley genome (Mascher et al., 2017) using the package ANNOVAR (Wang et al., 2010) (Version 2019-10-24). The pairwise linkage disequilibrium (R^2^) of SNPs in *HvSWEET* genes were calculated using PLINK (Purcell et al., 2007) (Version 1.90) and displayed using R package LDheatmap (Shin et al., 2006) (Version 1.0-6). The network 10 (https://www.fluxus-engineering.com/) was used to construct median-joining network of different haplotypes (Bandelt et al., 1999).

### Construction of vectors

Primers were designed according to open reading frames of *HvSWEET1a*, *HvSWEET4* and *AtSWEET1* genes. The corresponding sequences were amplified from cDNA of barley (Golden Promise) or Arabidopsis, and cloned into pDONR201 vector (Invitrogen). The clones were selected by PCR and sequenced to confirmation. For subcellular localization of HvSWEET1a and HvSWEET4 in tobacco leaves, the corrected pDONR201 vectors were recombinant with destination vectors pH7WGF2.0 to obtain GFP-HvSWEET1a and GFP-HvSWEET4 constructs using Gateway system. For complementation of yeast mutant EYB.VW4000 (Wieczorke et al., 1999), lacking 18 hexose transporters, and subcellular localization of SWEET proteins in yeast cells, the seamless cloning was used to introduce *SWEET* gene and *GFP* gene amplified from pFA6a-GFP(S65T)-His3MIX6 vector, into yeast expression vector pYEPlac195 vector, which was inserted by ADH1 promoter amplified from pADGT7 vector in advance, to obtain pADH1-SWEET-GFP(S65T) constructs. The primer information is listed in **Supplementary Table 1**.

### Subcellular localization in tobacco leaves

The subcellular localization vectors were transiently expressed in tobacco (*Nicotiana benthamiana*) leaves by Agrobacterium-mediated infiltration. AtPIP2A-meCherry was used as plasma membrane (PM) marker. The *Agrobaterium* strain C58C1 harboring p19 was used to prevent the onset of PTGS (post-transcriptional gene silencing) in the infiltrated leaves. Infiltrated tobacco plants were grown for another 3 days for GFP and mCherry imaging using a Zeiss LSM710NLO confocal laser-scanning microscope. Excitation/emission wavelength were 488 nm for GFP, and 561 nm for mCherry.

### Yeast mutant complementary growth assay and confocal microscope imaging

The yeast complementation vectors or empty vectors were transformed into the hexose-uptake deficient yeast mutant EYB4000 (Wieczorke et al., 1999). Then, transformed yeasts were screened in synthetic dropout (SD)-Ura media, supplemented with 2% maltose. For complementation growth assays, yeasts were grown overnight in liquid SD media to an optical density at 600 nm (OD_600_) of ~0.6, then OD_600_ was adjusted to ~0.3 with water. Five-microliter aliquots of serial dilutions were plated on SD media containing 2% maltose (as control) or 2% other hexoses. Growth photographs were taken after incubation at 30 °C for three days.

For subcellular localization of SWEET proteins in yeast cells, transformed yeasts cultured in SD media supplemented with 2% maltose were collected, washed three times with water, and then applied on microscope slide. Fluorescence signals were detected using a Zeiss LSM710NLO confocal laser-scanning microscope. Excitation/emission wavelength were 488 nm for GFP signal.

## Results

### Identification and phylogenetic analysis of *SWEET* genes in barley

A total of 23 *SWEET* genes were identified in barley, consistent with previously studies (Mascher et al., 2017; Qin et al., 2020), and were named accordingly (**Supplementary Table 2**). We analyzed the characteristics of *HvSWEET* genes, including exon number, protein length, the number of MtN3/saliva domain, molecular weight (MW) and isoelectric point (pI), and found that most proteins contained two MtN3/saliva domains and four contained only one domain (HvSWEET7b/c and HvSWEET15b/c), the protein length of HvSWEET ranged from 91 amino acid (aa; HvSWEET7b/c) to 353 aa (HvSWEET13a), the MW ranged from 10.19 kDa (HvSWEET7b/c) to 38.74 kDa (HvSWEET13a), and the pI ranged from 6.51 (HvSWEET12) to 11.3 (HvSWEET15c) (**Supplementary Table 2**).

**Figure 1:**
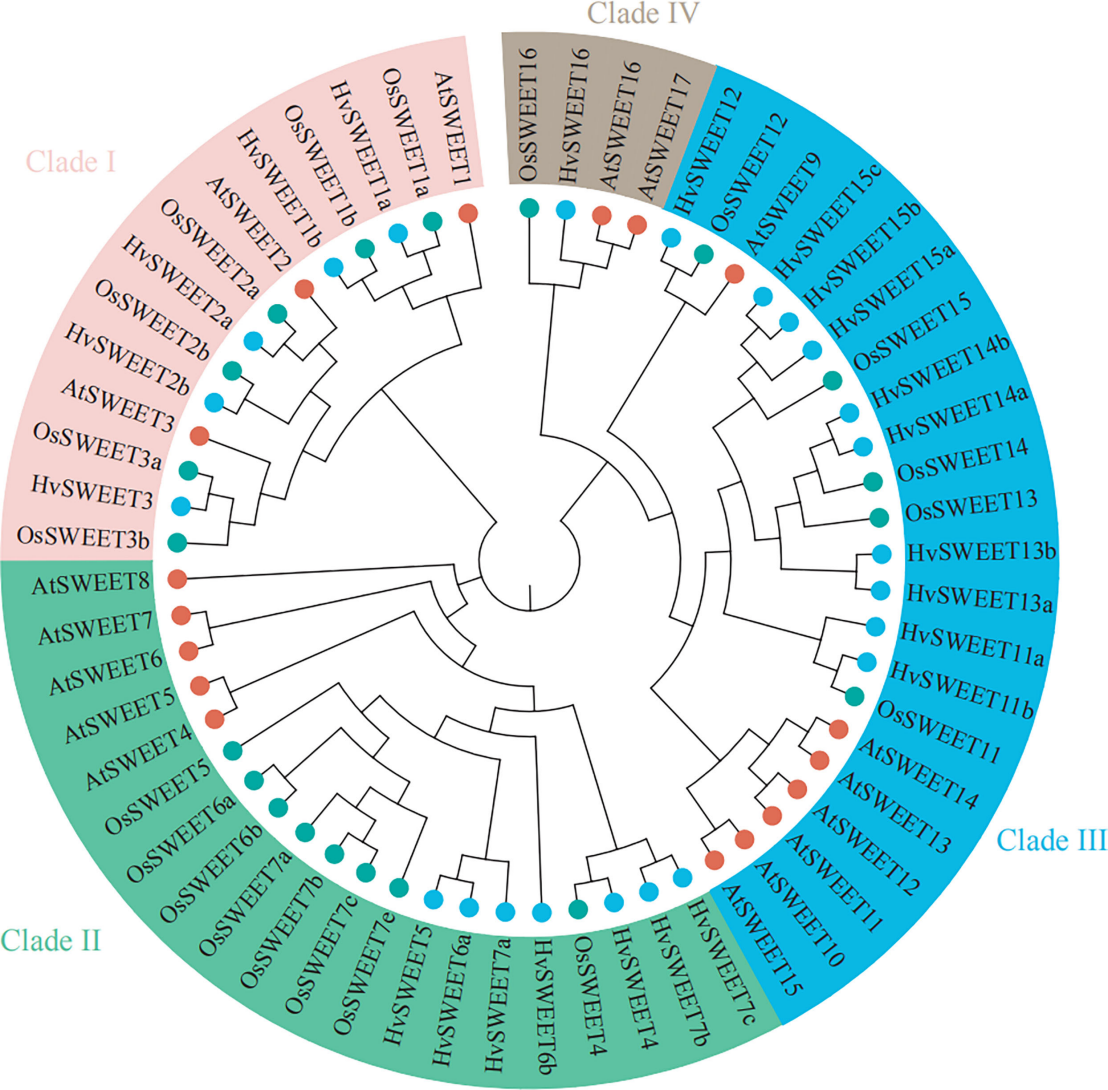
Phylogenetic relationship of SWEET family proteins of Arabidopsis, rice and barley. The phylogenetic tree is constructed using the neighbor-joining (NJ) method with 1000 bootstrap replications. Proteins of Arabidopsis, rice and barley are indicated with red, green and blue circles, respectively.

To investigate the evolutionary relationship between HvSWEET and SWEET proteins from other species, including Arabidopsis and rice, we constructed a neighbor-joining (NJ) phylogenetic tree and found that HvSWEET proteins could be clustered into four clades, with clade I containing five members (HvSWEET1a/b, HvSWEET2a/b and HvSWEET3), clade II containing seven members (HvSWEET4, HvSWEET5, HvSWEET6a/b and HvSWEET7a/b/c), clade III containing 10 members (HvSWEET11a/b, HvSWEET12, HvSWEET13a/b, HvSWEET14a/b, HvSWEET15a/b/c) and clade IV containing one member (HvSWEET16), respectively (**Figure 1**). A small extension of *HvSWEET11, HvSWEET13, HvSWEET14* and *HvSWEET15* were observed, with each containing two or three members in barley compared with only one orthologue in Arabidopsis and rice, consistent with previous study (Mascher et al., 2017).

### Duplication and synteny analysis of *HvSWEET* genes

**Figure 2:**
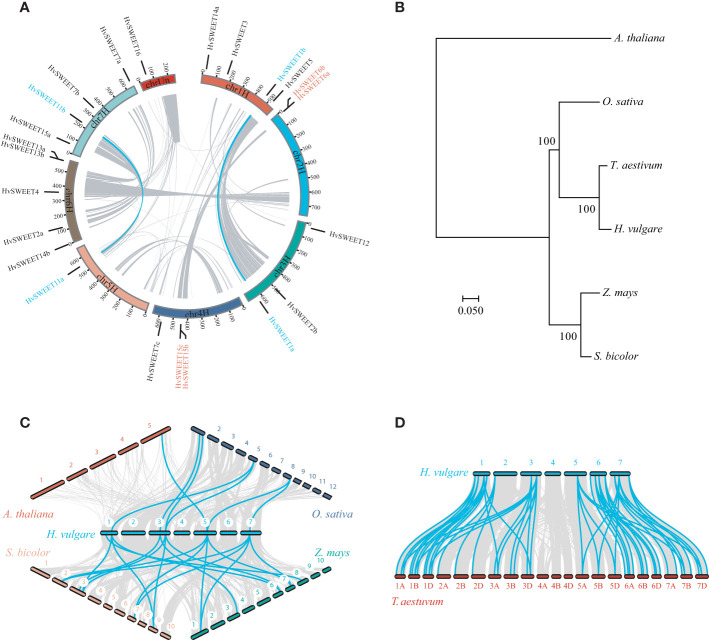
Snyteny analysis of *SWEET* family genes. **(A)** Snyteny analysis of *HvSWEET* genes within barley genome. The gray and blue lines in the inner circle denote syntenic blocks and duplications of *HvSWEET* gene pairs, respectively. The chromosome locations of *HvSWEET* genes are indicated with black lines linked with gene names, and segmental and tandem duplicated *HvSWEET* genes are texted in the blue and red colors, respectively. **(B)** Phylogenetic species tree of barley, Arabidopsis, rice, sorghum, maize and wheat using maximum-likelihood method with 1000 bootstrap replications. **(C)** and **(D)** Synteny analysis of *SWEET* family genes between barley and other plant species. The gray and blue lines represent snytenic blocks and homologous *SWEET* gene pairs, respectively.

Given that segmental and tandem duplications play important roles in gene family expansion during evolution, we detected these duplication events, involving *HvSWEET* genes, in barley. Two segmental duplications (*HvSWEET1a/b*, *HvSWEET11a/b*) and two tandem duplications (*HvSWEET6a/b*, *HvSWEET15b/c*) were observed (**Figure 2A**). Additionally, *HvSWEET* genes were observed uneven distributed over barley chromosomes, with chromosome 6 containing five members, chromosome 1 and 7 each containing four members, chromosome 3 and 4 each containing three members, chromosome 2 containing two members, and chromosome 5 and chromosome unscaffold each containing one member, respectively (**Figure 2A**).

To explore the evolution relationships of *SWEET* family genes between barley and other plant species, we performed synteny analysis between barley and Arabidopsis or Poaceae plant species, including rice, maize, sorghum and wheat. While only one pair of orthologous *SWEET* genes was observed between barley and Arabidopsis, which might be caused by evolutionally far genetic relationship between dicots and monocots (**Figure 2B**), there were six, eight, nine and 51 pairs of orthologous genes identified between barley and rice, maize, sorghum and wheat, respectively (**Figures 2C, D**). The closer evolution relationship and high ploidy levels of wheat might lead to more syntenic orthologous *SWEET* gene pairs between barley and wheat (**Figure 2B**).

### *HvSWEET* genes structures analysis

**Figure 3:**
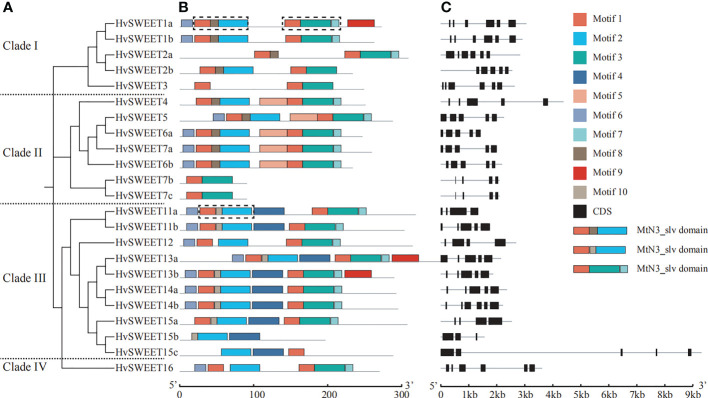
Phylogenetic relationship, conserved protein motifs and gene structures of *HvSWEET* genes. **(A)** Phylogenetic relationship analysis of *HvSWEET* genes using the neighbor-joining (NJ) method with 1000 bootstrap replications. **(B)** Conserved motif analysis of HvSWEET proteins. Different motifs are indicated with different colored boxes. The MtN3_slv domain is outlined using black dashed lines. **(C)** Gene structures of *HvSWEET* genes. The black boxes represent CDS regions.

To gain more insights into the characteristics of *HvSWEET* genes, the gene structural diversity was examined, and 11 members were found to have five exons, six members six exons, five members four exons and one member three exons (**Figure 3C**). The exon lengths were similar, whereas the intron lengths varied, with *HvSWEET4*, *HvSWEET15c* and *HvSWEET16* containing very long introns (**Figure 3C**). Furthermore, we found clade I members contain six exons except *HvSWEET2b* containing five exons, clade II members contain five exons except *HvSWEET7b* and *HvSWEET7c* containing four exons, clade IV member contains six exons, and clade III members contain three to six exons (**Figures 3A–C**), suggesting that the gene structures of clade I and clade II are relatively conserved.

To further investigate the gene structural diversity, the conserved motifs of all HvSWEET proteins were examined. In total, ten conserved protein motifs, with the range from eight to 41 aa (**Supplementary Figure 1**), were identified, among which the group containing motif 1, 8/10 and 2 and the group containing motif 1, 3 and 7 were annotated as MtN3_slv domain (**Figure 3B**). In addition, the result demonstrated that the motif 1, 2, 3, 6 and 7 exist in most HvSWEET members (96%, 83%, 91%, 61% and 74%, respectively), and motif 4 was identified only in the clade III. The HvSWEET protein in the same clade share relatively similar conserved motifs.

### Tissue expression patterns of *HvSWEET* genes

**Figure 4:**
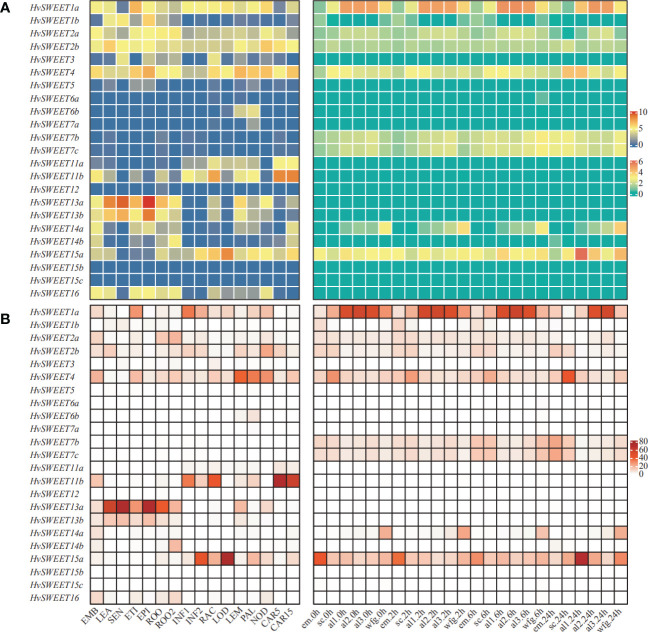
Expression profiles **(A)** and expression proportions of *HvSWEET* genes in that of the whole gene family **(B)** in the published different developmental tissues (Mascher et al., 2017) (left panel) and seed tissues during germination (Betts et al., 2017) (right panel). The TPM values of each gene, normalized by TMM method, are used for **(B)** and the transformation values by log_2_ (TPM+1) are used for **(A)**. Left panel: EMB, 4 days after planting (dap) embryo; LEA, 17 dap leaf; SEN, 56 dap senescing leaf; ETI, 10 dap etiolated leaf grown in dark; EPI, 28 dap epidermal strips; ROO, 17 dap root; ROO2, 28 dap root; INF1, 30 dap inflorescences; INF2, 50 dap inflorescences; RAC, 35 dap inflorescence rachis; LOD, 42 dap lodicule; LEM, 42 dap lemma; PAL, 42 dap palea; NOD, the third stem internode (42 dap); CAR5, 5 days post-anthesis (dpa) caryopsis; CAR15, 15 dpa caryopsis. Right panel: em, embryo; sc, scutellum; al1, one third of aleurone proximal to the embryo; al2, the central third of aleurone; al3, one third of aleurone distal to embryo; wfg; whole fixed grain; 0h, 2h, 6h and 24h represent the corresponding time after seed germination.

To comprehensively study the physiological functions of *HvSWEET* genes, the raw RNA-seq sequencing data of 16 different developmental tissues and seed tissues during germinating were derived from previously study (Betts et al., 2017; Mascher et al., 2017), and used to map against barley reference genome (Mascher et al., 2017). Different expression patterns were observed for *HvSWEET* genes in the detected tissues (**Figure 4A**). Five *HvSWEET* genes, including *HvSWEET1a*, *HvSWEET2a/b*, *HvSWEET4* and *HvSWEET15a*, were expressed in almost all detected tissues, whereas four *HvSWEET* genes, including *HvSWEET5*, *HvSWEET12* and *HvSWEET15b/c*, were nearly not detected (**Figure 4A**; **Supplementary Tables 3, 4**). Moreover, several *HvSWEET* genes were expressed during seed germination stages, such as *HvSWEET7b/c*, and several genes showed high expression levels in specific tissues, such as *HvSWEET11b* in developing caryopsis and inflorescence rachis, and *HvSWEET13a/b* in senescing leaf and epidermal strips (**Figure 4A**; **Supplementary Tables 3, 4**). Additionally, we examined the expression patterns of duplicated *HvSWEET* gene pairs, and found that some duplicated genes exhibited divergent expression patterns, such as *SWEET1a/b* and *HvSWEET11a/b*, suggesting they underwent neofunctionalization after duplications (**Figure 4A**; **Supplementary Tables 3, 4**).

Given the functional redundancy of *HvSWEET* genes, the relative expression level determines which member plays the main role in sugar transporting in the specific tissue within the gene family. We calculated proportion of each *HvSWEET* gene expression in that of the whole family, and observed that the sugar transporting activities of the gene family in leaf, senescing leaf, epidermal strips and root were mainly dependent on *HvSWEET13a*, the inflorescence, rachis and lodicule mainly dependent on *HvSWEET11b* and *HvSWEET15a*, the lemma and palea mainly dependent on *HvSWEET4*, the developing caryopsis mainly dependent on *HvSWEET11b*, and the germinating seed aleurone, embryo and scutellum mainly dependent on *HvSWEET1a*, *HvSWEET15a* and *HvSWEET4*, respectively (**Figure 4B**). These results demonstrate *HvSWEET* genes function redundantly and divergently to mediate sugar transport in barley developmental growth.

### *HvSWEET1a* was artificial selected during barley domestication and improvement

**Figure 5:**
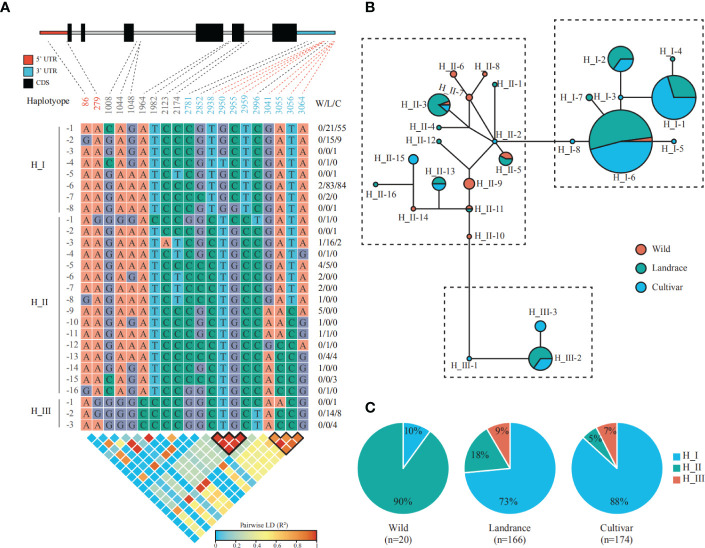
Artificial selection of *HvSWEET1a* gene during barley domestication and improvement. **(A)** Genetic variations detected in the genomic region of *HvSWEET1a* in the published exome re-sequencing data of 360 accessions (Bustos-Korts et al., 2019). The SNP positions are indicated relative to the gene start site of *HvSWEET1a*, 634,920,942 on chromosome 3, and displayed below gene structure, linked with dashed lines. The red dashed lines and black outlines denote the SNPs in strong LD regions in top and bottom panel, respectively. The W/L/C indicates the number of wild accessions, landraces and cultivars, respectively. The pairwise linkage disequilibria (LD) between SNPs are measured using R^2^. **(B)** Median-joining network representing the relatedness of 27 *HvSWEET1a* haplotypes, each represented by a circle with size proportional to the corresponding haplotype population size. Wild accessions, landraces and cultivars are indicated by red, green and blue colors, respectively. **(C)** Allele frequency distribution of three main haplotypes. H_I, blue; H_II, green; H_III, red. The accession number (n) is shown below the corresponding population.

Barley was selected by early humans in the Fertile Crescent ground 10,000-12,000 years ago and is primarily used for animal feed, and malting and brewing, with a small percentage devoted to human food (Stein and Muehlbauer, 2018). The uniform and fast germination is a typical domestication syndrome trait, which ensure even maturity and enable crop management (Fuller and Allaby, 2009). Moreover, high malting quality is of importance for barley and also requires uniform and fast seed germination, during which the malting grains develop the enzymes required for modifying starches into various types of sugar to fuel the embryonic axis growth (Ma et al., 2017). Given overexpression of the sugar carrier *AtSWEET16* resulted in improved seed germination rates (Klemens et al., 2013), we thought *HvSWEET1a* and *HvSWEET4*, relatively high expressed in aleurone and scutellum during seed germination, respectively, might be related with seed germination and could undergo artificial selection during barley domestication (wild accessions versus landraces) and improvement (landraces versus cultivars) (**Figure 4B**). To verify our hypothesis, we detected the genetic variations of *HvSWEET1a* using the exome SNP information of 360 accessions, consisting of 20 wild accessions, 166 landraces and 174 cultivars, from previously study (Bustos-Korts et al., 2019). In total, 20 SNPs, consisting of two SNPs in 5’ UTR, seven SNPs in introns and 11 SNPs in 3’ UTR, were detected in *HvSWEET1a* genetic region and classified the population into 27 haplotypes, each represented by one to more than one hundred accessions (**Figure 5A**). Median-joining network analysis sorted the 27 haplotypes into three main groups, namely H_I group (including H_1-1 to H_I-8), H_II group (including H_II-1 to H_II-16) and H_III group (including H_III-1 to H_III-3) (**Figure 5B**). Allele-frequency analysis demonstrated that the proportion of H_I significantly increased in landraces compared with that of wild accessions, and further increased in cultivars, indicating strong artificial selection of *HvSWEET1a* during barley domestication and improvement (**Figure 5C**). In addition, two groups of SNPs in the 3’ UTR region of *HvSWEET1a* gene showed strong linkage disequilibrium (LD), which might be the artificial selected regions (**Figure 5A**).

Similarly, the genetic variations of *HvSWEET4* were also examined and nine SNPs were detected, which sorted the population into 12 haplotypes (**Supplementary Figure 2**). Median-joining network sorted the 12 haplotypes into three main groups, namely H_I group (including H_I-1), group H_II (including H_II-1 to H_II-8) and group H_III (including H_III-1 to H_III-3) (**Supplementary Figure 2**). However, no significant allele-frequency variations of three main groups were observed among wild accessions, landraces and cultivars (**Supplementary Figure 2**).

### HvSWEET1a and HvSWEET4 have transport activity of hexoses

**Figure 6:**
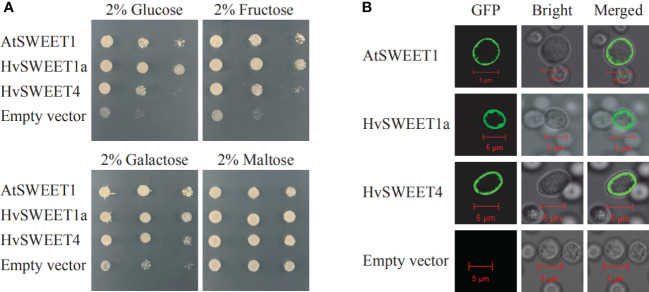
HvSWEET1a and HvSWEET4 have transport activity of hexoses. **(A)** Complementation of hexose uptake-deficiency of the yeast mutant EYB.VW 4000. Glucose, Galactose, Fructose and Maltose denote the corresponding carbon source used in the media. Photographs were taken 3 days after incubation. Experiment was repeated three times with similar results. AtSWEET1, positive control; Empty vector, negative control. **(B)** Plasma membrane subcellular localizations of HvSWEET1a, HvSWEET4 and AtSWEET1 in yeast cells. Bars = 5 μm.

To validate the sugar transporter function of HvSWEET1a and HvSWEET4 in yeast, the N-terminal HvSWEET1a or HvSWEET4 in fusion with GFP were transformed into hexose-uptake deficient yeast EYB.VW4000 (Wieczorke et al., 1999), which lacks 18 hexose transporters and could not grow on media with hexose as the only carbon source, with AtSWEET1 as the positive control. The results revealed that the plasma membrane (PM) localized HvSWEET1a and HvSWEET4 could transport glucose, galactose and fructose in yeast cells, and HvSWEET1a showed higher transport activity than HvSWEET4 (**Figures 6A, B**). To study the subcellular localization of HvSWEET1a and HvSWEET4 in plant cells, GFP fused with HvSWEET1a or HvSWEET4 was co-expressed with Arabidopsis PM intrinsic protein 2A fused to mCherry (AtPIP2A-mcherry) (Prak et al., 2008) in tobacco leaves. GFP fluorescence signals of HvSWEET1a or HvSWEET4 coincided to mCherry signals of PM maker in tobacco leaves (**Supplementary Figure 3**). These results demonstrate that HvSWEET1a and HvSWEET4 function as PM hexose transporters.

## Discussion

Sucrose is produced in photosynthetically active tissues of the leaf and stem and actively loaded into phloem, and then translocated to various tissues and cells that depend on external sugar supply. SWEET transporters play vital roles in the sugar transport process (Eom et al., 2015), especially in the angiosperm grain filling, where only apoplastic pathway exists between maternal and filial tissues including endosperm and embryo (Yang et al., 2018). Genome-wide identification and analysis is an essential method to study the specific functions of the gene family. However, high content of repetitive elements and large pericentromeric regions lead to relatively late publication of chromosome-level barley reference genome (Mascher et al., 2017), hindering the gene family identification compared with other plant species (Chen et al., 2010; Patil et al., 2015; Xie et al., 2019; Zhang et al., 2019; Qin et al., 2020). The barley *SWEET* family members were identified in previously study (Mascher et al., 2017; Qin et al., 2020), but they were not systematically investigated. In this study, we comprehensively analyzed the barley *SWEET* family. We carried out genome-wide identification of *HvSWEET* genes and found 23 members, consistent with previously study (Mascher et al., 2017; Qin et al., 2020). The barley *HvSWEET* members were clustered into four clades, with a litter family member expansions (*HvSWEET11, HvSWEET13, HvSWEET14* and *HvSWEET15*) compared with Arabidopsis and rice (**Figure 1**). The *HvSWEET* genes in the same clade shared relatively similar gene structures and conserved protein motifs (**Figure 3**). Moreover, two tandem and segmental duplicated *HvSWEET* gene pairs were observed in the barley genome, and the phylogenetic species relationship and polyploidization might account for the differences of *SWEET* gene pairs identified between barley and other plant species (**Figure 2**). *HvSWEET* genes showed different expression patterns (**Figure 4A**), indicative of their divergent functions in various biological processes. Interestingly, different expression patterns of duplicated *HvSWEET* genes were observed, demonstrating that gene neofunctionalization occurred after duplications (**Figure 4A**). Considering functional redundancy, we also examined the proportion of single *HvSWEET* gene in that of the whole family to identify the main functional member in specific tissue and observed the relatively high expression of *HvSWEET1a* and *HvSWEET4* in seed aleurone and scutellum during germination, respectively (**Figure 4B**). Given seed germination is a typical domesticated trait and *SWEET* genes is related with seed germination rates (Klemens et al., 2013), we performed haplotype analysis and observed the artificial selection of *HvSWEET1a* during domestication and improvement (**Figures 5**, **6**). Considering the SNP positions and LD values, the 3’ UTR of *HvSWEET1a* might be the region under selection (**Figure 5A**). Finally, the yeast complementary experiment and subcellular localization demonstrated that HvSWEET1a is a PM hexose transporter (**Figure 6**; **Supplementary Figure 3**). Although it is plausible to infer that *HvSWEET1a*, highly expressed in aleurone tissue during seed germination, was artificial selected due to facilitate seed germination, we could not exclude the possibilities that *HvSWEET1a* underwent selection for other important traits, considering the tissue expression profile of *HvSWEET1a* (**Figure 4**), and further experiments are needed to investigate the physiological function and the domestication mechanism of *HvSWEET1a*.

The reference genome assembly quality plays an important role in gene family identification and the recent development of fast and accurate long-read sequencing by circular consensus sequencing (CCS) facilitated the barley reference genome assembly version 3 (Mascher et al., 2021). To compare our genome-wild identification result of *HvSWEET* gene family, which was based on barley reference genome version 1 (Mascher et al., 2017), with that based on the version 3 (Mascher et al., 2021), we performed the same methods to identify *HvSWEET* family genes in the version 3. Surprisingly, 26 *HvSWEET* members were observed in version 3 with three more members (**Supplementary Table 5**). The phylogenetic analysis also classified the 26 members into four clades (**Supplementary Figure 4**). To investigate whether differences of *HvSWEET* genes exist between version 1 and version 3, we compared the protein similarity, exon numbers and MtN3/Saliva domain numbers of the two version members, and found that almost all the *HvSWEET* genes (version 1) share the same exon numbers and MtN3/Saliva domain numbers with corresponding ones in version 3 (**Supplementary Table 5**), and the majority (18/23) of *HvSWEET* genes (version 1) shared 100% similarity with those in version 3, four members more than 90% and one member more than 80% (**Supplementary Table 5**). Interestingly, *HvSWEET16*, which is located in chromosome unscaffold in version 1, is relocated to chromosome 5 in version 3 (**Supplementary Table 5**). The scaffold N50 length of barley reference genome version 3 is more than 60 times of that version 1 (Mascher et al., 2017; Mascher et al., 2021), and the better assembly quality and gene annotations account for more *HvSWEET* family members identified and chromosome location of *HvSWEET16* in version 3. In the near future, more and more omics data based on the barley reference genome version 3 will further facilitate our understanding of *HvSWEET* gene family, especially for the newly identified *HvSWEET* members.

Crop domestication is one of the most significant innovations in the history of humankind, which has enabled humans to survive, multiply, and ultimately give birth to the development of civilization (Doebley et al., 2006). However, the genetic diversities of modern crops significantly decreased compared with their progenitors for long term domestication and improvement (Huang et al., 2012; Hufford et al., 2012; Zhou et al., 2015; Milner et al., 2019), making them lack diversities in important genes, such as stress-resistance genes, and vulnerable to the rapid climate changes. More importantly, it is very difficult to develop super varieties based on the modern germplasms to meet the globally increasing food demand in face of extreme environmental challenges. Rapid *de novo* domestication of wild species is an alternative breeding strategy and has been successful in a few plant species by introducing desired mutations in specific genes, which were under selection pressure during domestication, in wild background with the powerful genome editing technology CRISPR/Cas9, and the elite lines with domestication traits and stress-resistance were obtained in a few years (Li et al., 2018; Zsogon et al., 2018; Yu et al., 2021). The pathogen, *Xanthomonas oryzae pv. oryzae* (*Xoo*), secretes transcription-activator-like effectors (TALes) to recognize effector-binding elements (EBEs) and induce, at minimum, one of *OsSWEET11*, *OsSWEET13* and *OsSWEET14* to increase sugar content in the invasion sites, and simultaneous introduction of mutations in EBE regions of all three *OsSWEET* promoters using CRISPR/Cas9 in rice line Kitaake and elite mega varieties IR64 (Mackill and Khush, 2018) and Ciherang-Sub1 (Toledo et al., 2015) confers them robust and broad-spectrum resistance to rice blight (Eom et al., 2019; Oliva et al., 2019). The promoter of maize *ZmSWEET4c* was strongly selected during domestication and the higher gene expression in maize than maize ancestor teosinte leads to larger grains (Sosso et al., 2015). The artificial selection of a 9-base pair deletion in the promoter of soybean *GmSWEET10a* during domestication and improvement upregulates the gene expression and results in higher oil content (Miao et al., 2020; Wang et al., 2020). Priority of *De novo* domestication is functional investigations of artificial selected genes, and our study provides a potential candidate gene for *de novo* domestication breeding of barley.

## Conclusion

In this study, a total of 23 barley *HvSWEET* genes were identified, with two tandem and two duplicated, and divided into four clades. Most genes belonging to the same clade showed similar gene structures and conserved motifs. Expression patterns of *HvSWEET* genes varied, and *HvSWEET1a* and *HvSWEET4*, highly expressed in seed aleurone and scutellum during germination, respectively, showed PM subcellular localization and hexose transport activity. Moreover, haplotype analysis revealed the artificial selection of *HvSWEET1a* during barley domestication and improvement. The obtained results will be helpful for comprehensive understanding and further functional investigations of barley *HvSWEET* family, and also provide a potential candidate gene for *de novo* domestication breeding of barley.

## Data availability statement

The datasets presented in this study can be found in online repositories. The names of the repository/repositories and accession number(s) can be found in the article/**Supplementary Material**.

## Author contributions

JW, WY, and KC conceived and designed the research. WY and KC performed data analysis. XX and LL carried out the experiment. WY and JW wrote the manuscript. All authors contributed to the article and approved the submitted version.
