# Supplementary figures and images for: Genome-wide identification, expression pattern and genetic variation analysis of SWEET gene family in barley reveal the artificial selection of HvSWEET1a during domestication and improvement
Source: Front Plant Sci. 2023 Feb 13;14:1137434. doi: 10.3389/fpls.2023.1137434 (PMC9968841; doi:10.3389/fpls.2023.1137434)

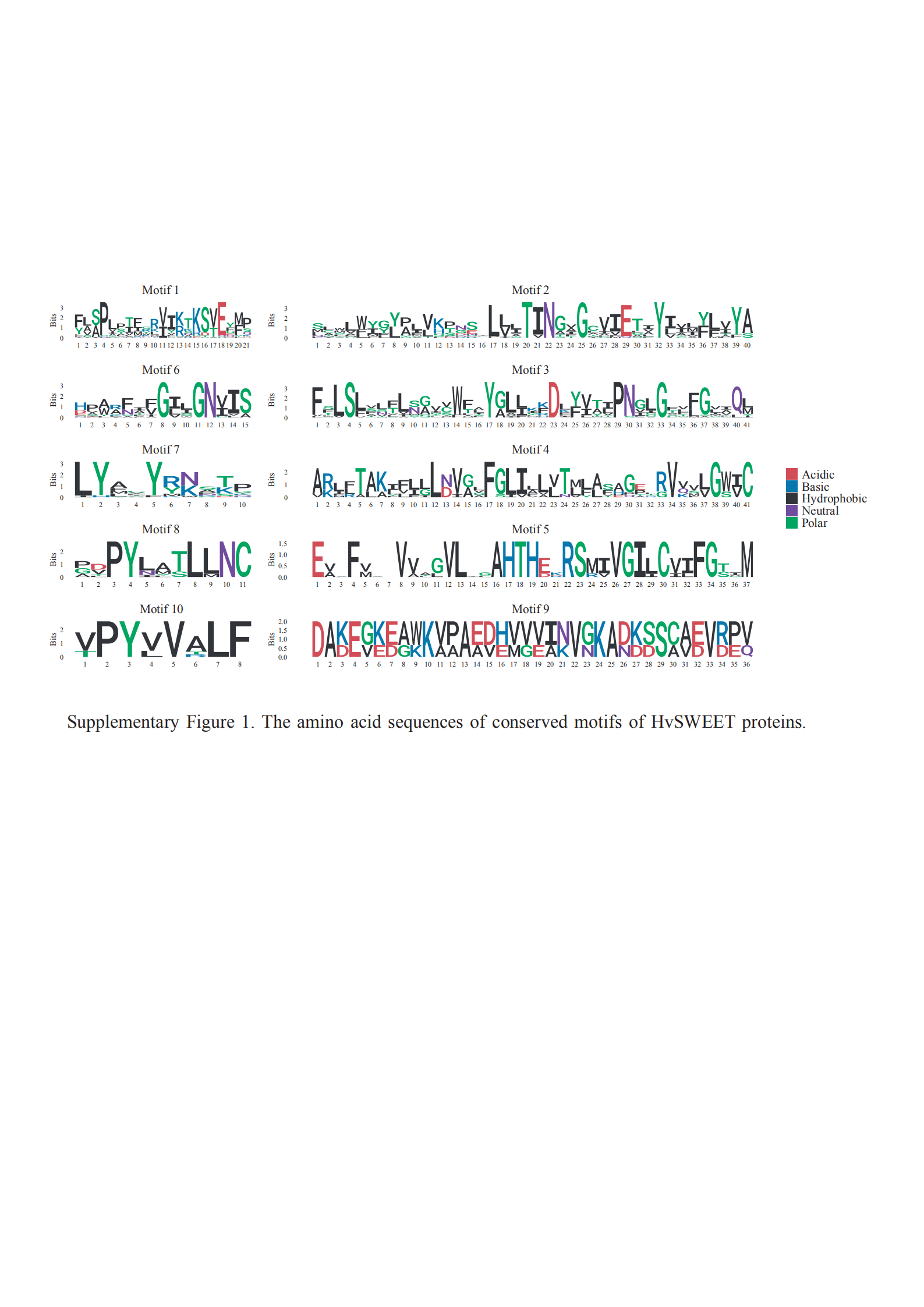

Supplement: Supplementary file 1 [file Image_1.tif]

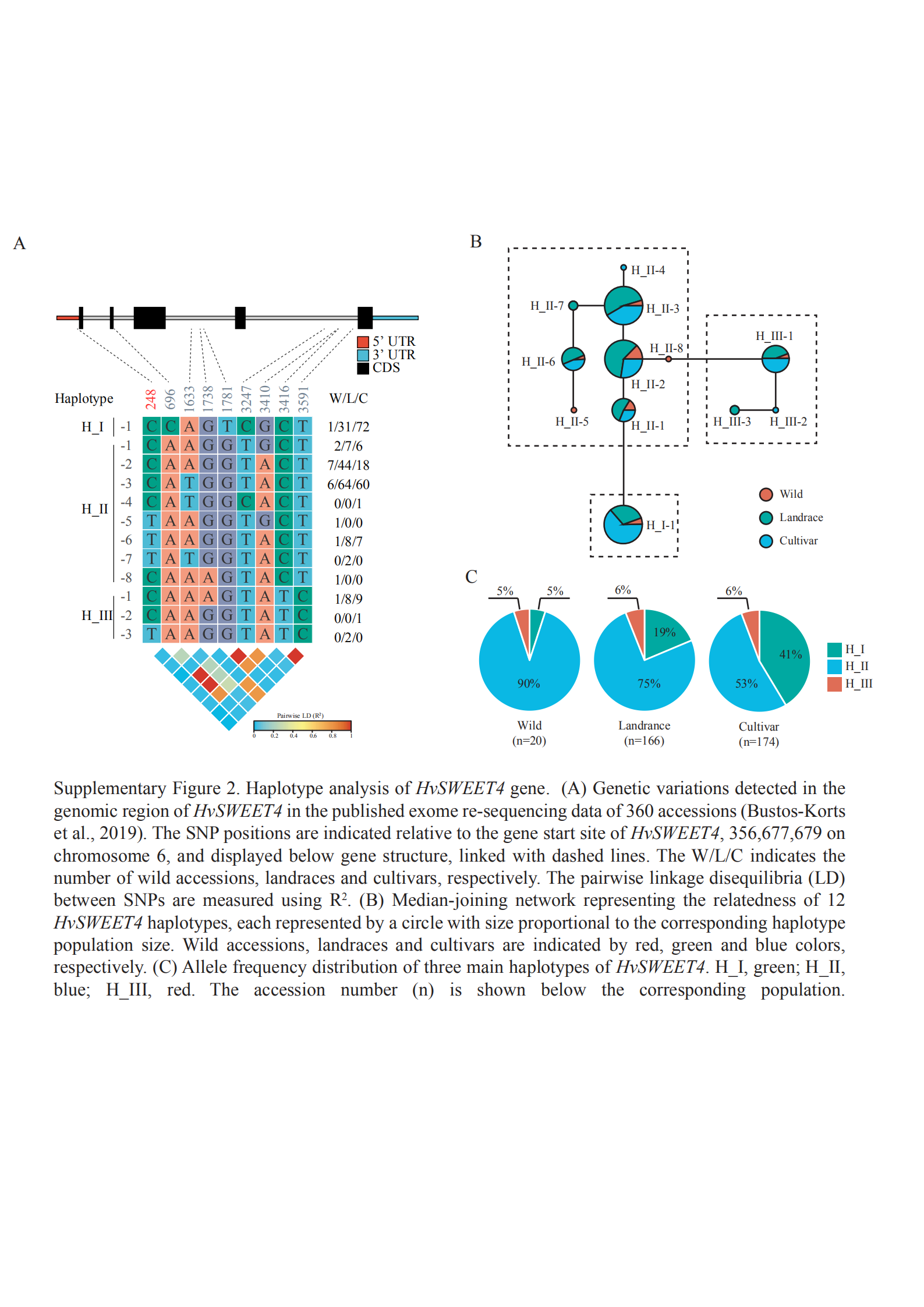

Supplement: Supplementary file 2 [file Image_2.tif]

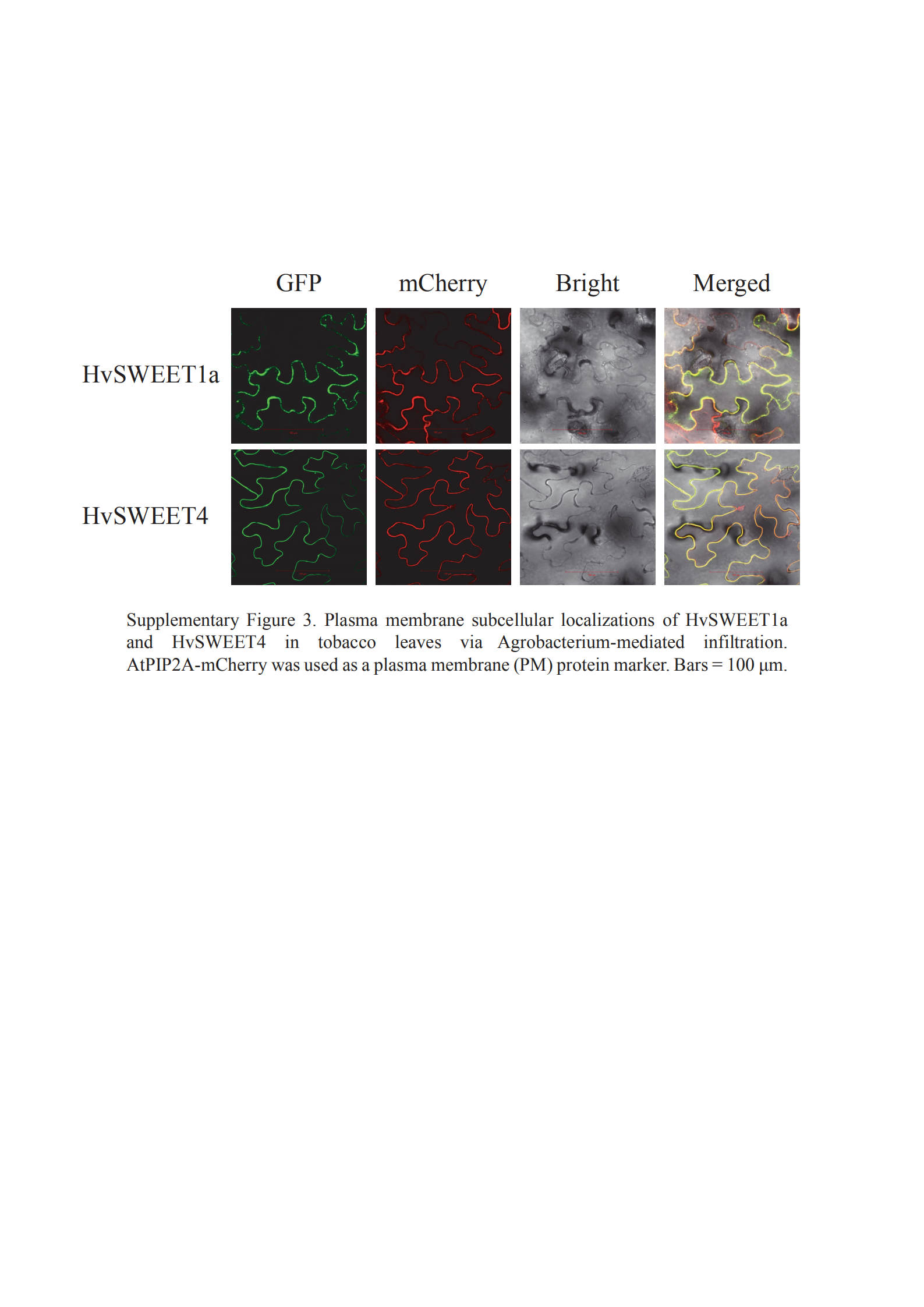

Supplement: Supplementary file 3 [file Image_3.tif]

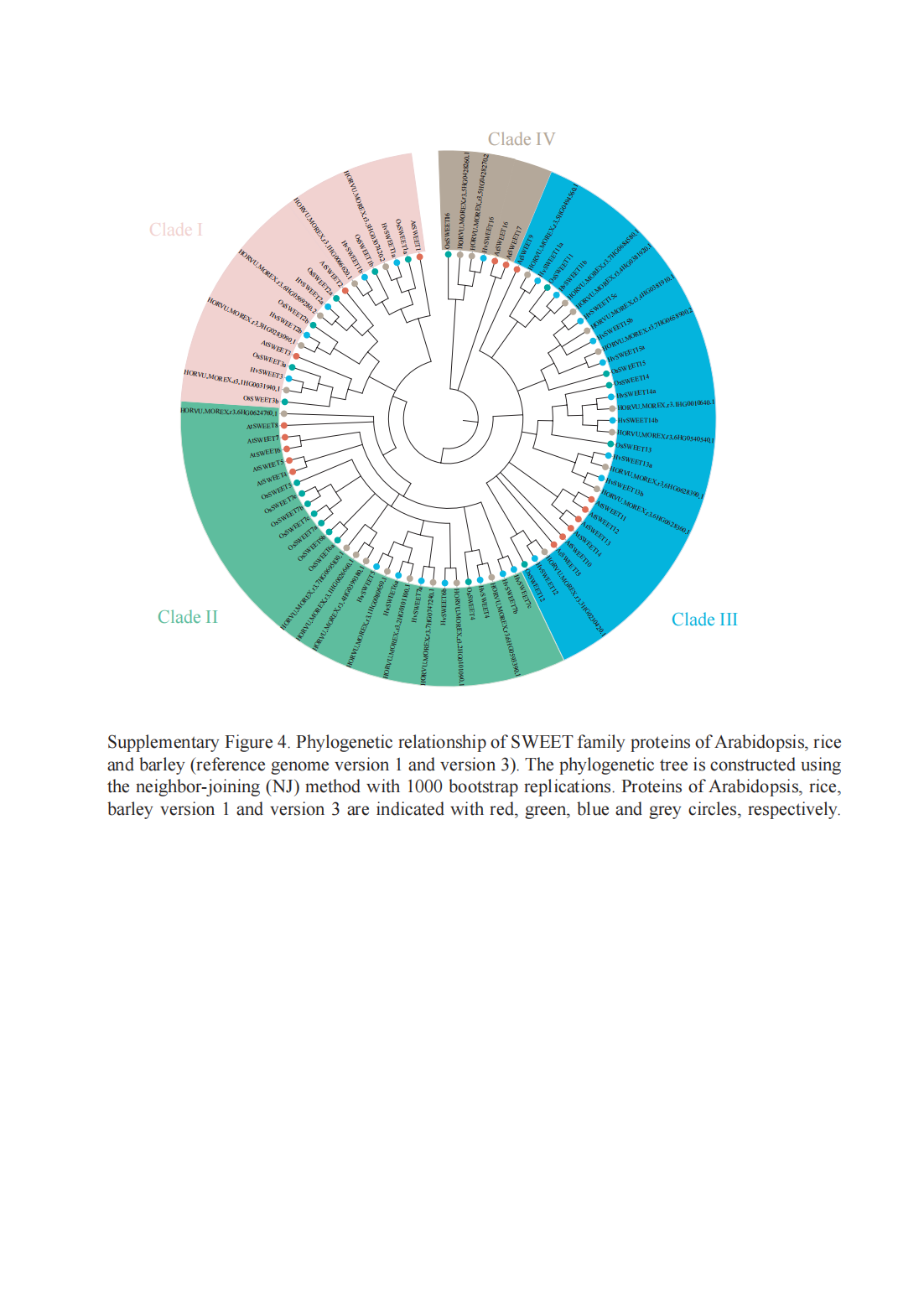

Supplement: Supplementary file 4 [file Image_4.tif]
